# An alignment-free method for detection of missing regions for phylogenetic analysis

**DOI:** 10.1016/j.heliyon.2024.e32227

**Published:** 2024-06-04

**Authors:** Rubyeat Islam, Atif Rahman

**Affiliations:** a Department of Computer Science and Engineering, Military Institute of Science and Technology, Dhaka, Bangladesh; b Department of Computer Science and Engineering, Bangladesh University of Engineering and Technology, Dhaka, Bangladesh

**Keywords:** Phylogeny, Alignment-free, Missing regions, k-mer

## Abstract

Phylogenetic tree estimation using conventional approaches usually requires pairwise or multiple sequence alignment. However, sequence alignment has difficulties related to scalability and accuracy in case of long sequences such as whole genomes, low sequence identity, and in presence of genomic rearrangements. To address these issues, alignment-free approaches have been proposed. While these methods have demonstrated promising results, many of these lead to errors when regions are missing from the sequences of one or more species that are trivially detected in alignment-based methods. Here, we present an alignment-free method for detecting missing regions in sequences of species for which phylogeny is to be estimated. It is based on counts of *k*-mers and can be used to filter out *k*-mers belonging to regions in one species that are missing in one or more of the other species. We perform experiments with real and simulated datasets containing missing regions and find that it can successfully detect a large fraction of such *k*-mers and can lead to improvements in the estimated phylogenies. Our method can be used in *k*-mer based alignment-free phylogeny estimation methods to filter out *k*-mers corresponding to missing regions.

## 1 Introduction

Inference of phylogenetic trees i.e. trees that depict evolutionary relationships among species is one of the fundamental problems in computational biology. All life on earth is related through a single phylogenetic tree and the species that are closely related to each other have more similarities than those that are distantly related. While morphological traits were used to construct phylogenies in the past, it is primarily done using genomic sequences at present.

Phylogeny reconstruction methods can be broadly classified into two types, namely, distance based and character based. Distance based methods are reliant on the construction of a distance matrix in the first step, and generally, they require alignment of sequences from each pair of species whereas character based approaches need a multiple sequence alignment (MSA) of the sequences from all the species. Distance based algorithms such as the unweighted pair group method using arithmetic averages (UPGMA) [1] and Neighbor Joining (NJ) [2] have been widely used for phylogeny estimation over the years. On the other hand, maximum likelihood [3] and maximum parsimony [4] are well known approaches in the character based paradigm. In these methods, phylogenetic trees are estimated from a character matrix constructed using multiple sequence alignment.

However, sequence alignment is difficult to scale to large sequences, especially to whole genomes [5], sometimes leads to inaccuracies in case of low sequence identity, consumes a substantial amount of time and memory [6], and is hard to apply in the presence of sequence rearrangements [7].

To overcome these issues, alignment-free approaches have been proposed for phylogeny estimation that have offered noticeable advances in phylogenetics. In a comprehensive study [8], Haubold presented a classification of phylogeny construction methods and extensively reviewed alignment-free methods. Alignment-free methods rely primarily on partition or distance. But the partition approach fails to outperform simpler distance-based strategies [8], [9]. The distance-based approaches can be further classified into two types. The word count based approaches use counts of words of some fixed length whereas match length based ones utilize the lengths of matches between pairs of sequences. In alignment-free methods, distances are calculated using a number of approaches such as word or *k*-mer counts [10], lengths of common substrings [11], and micro-alignments [12]. Many widely used alignment-free approaches are based on counts of *k*-mers [13] i.e. contiguous sequences of length *k*. A number of methods have been devised to estimate the distance between a pair of species from the *k*-mer count vectors of the pair [8] (see Methodology). Moreover, several methods have been developed based on *k*-mer count for classification of viruses [14], metagenomic analysis [15], classification of taxa in environmental genomic data [16], and so on.

A challenge that arises in phylogeny estimation from long genomic sequences is due to missing data. While missing data can come in various forms in phylogeny estimation, here we are concerned with regions missing from sequences of one or more species which may happen due to erroneous or incomplete assemblies among other reasons. Using large-scale simulated or empirical data, the consequences of missing data on phylogenetic inference and approaches to deal with them are being explored [17], [18], [19], [20], [21], [22]. It has been observed that randomly distributed missing data have no effects in phylogenetic reconstruction while the consequence is just the opposite in the context of nonrandomly distributed missing data [17]. A number of studies have shown the robustness of methods to missing data and demonstrated that including taxa with missing data can lead to better accuracy than excluding them [18], [19], [20], [23].

**Figure 1:**
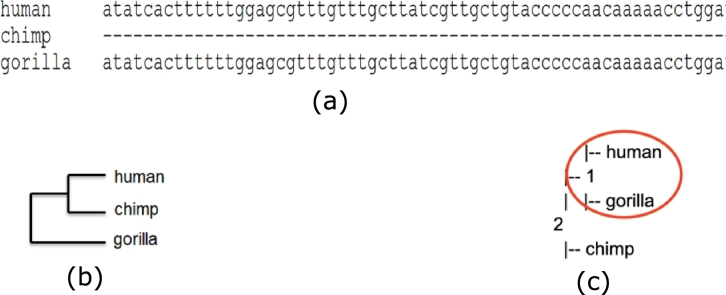
(a) Snapshot of a multiple sequence alignment of a human, chimpanzee and gorilla dataset, (b) The reference phylogenetic tree for the 3 species, and (c) The constructed phylogeny with missing regions for the 3 species.

However, the methods for phylogenetic inference that deal with missing regions are mainly alignment-based. In the alignment-free setting, missing regions are difficult to detect and can lead to incorrect phylogenies. For example, a large segment was found to be absent in a region of interest in the chimpanzee sequence collected from NIH Intramural Sequencing Center (NISC). Fig. 1(a) shows a snapshot of an MSA of human, chimpanzee, and gorilla where the missing segment appears as a sequence of gap characters (‘-’). While the missing region is trivially detected and ignored in alignment-based methods, this remains undetected in a number of alignment-free methods. As a consequence, the distances from chimpanzee to both human and gorilla are overestimated under a wide variety of distance calculation approaches. This leads to the incorrect phylogeny shown in Fig. 1(c) compared to the reference tree in Fig. 1(b). This illustrates the importance of detection of missing regions in phylogeny estimation which, to the best of our knowledge, has not been explored in an alignment-free setting.

Here we present an alignment-free approach for detecting missing regions. The method identifies *k*-mers that are likely to be from a missing region in one or more species and filters them out. We test our method on real and simulated datasets, and find that our method is able to detect and filter out large numbers of *k*-mers from missing regions. Moreover, phylogenetic trees constructed from the remaining *k*-mers tend to be more accurate than those constructed with all *k*-mers.

## 2 Methodology

In this section, we present the methodologies used in this article. First, we provide a general overview of an alignment-free distance based method using *k*-mers, and then discuss our method to detect and filter out *k*-mers from missing regions.

### 2.1 Overview

**Figure 2:**
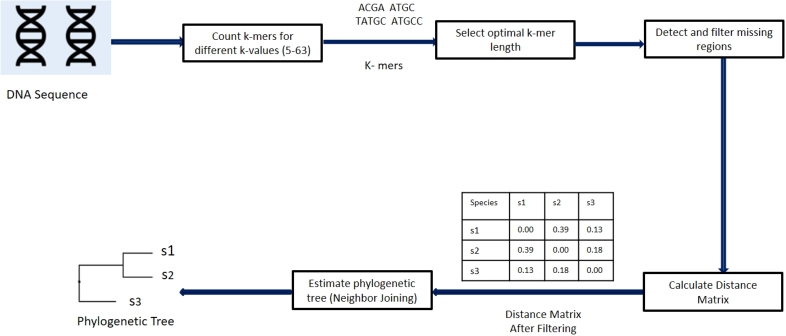
At first, a *k*-mer counting tool is used to generate *k*-mers with their frequencies from input DNA sequences. Then, the optimal *k*-mer length is selected based on some criterion. After this, we detect *k*-mers from missing regions. After filtering these *k*-mers, distance matrix is constructed and a phylogenetic tree is estimated using the Neighbor Joining algorithm.

Generally, in a distance based alignment-free approach using *k*-mers, first a *k*-mer counting tool is used to count *k*-mers in the sequences of the species. An issue that often arises is the selection of the optimal value of *k*. Then the *k*-mer counts are used to construct a distance matrix containing estimated distances between each pair of species. A distance based phylogeny estimation method such as Neighbor Joining is finally used to construct a phylogeny from the distance matrix. However, if there are missing regions in the sequences, the distances calculated may be inaccurate. So, we add a ‘Detect and filter missing regions’ step in our methodology to identify and filter out *k*-mers corresponding to missing regions in one or more species before distance matrix construction and phylogeny estimation. Fig. 2 shows the full process of phylogenetic reconstruction used in this research.

### 2.2 *k*-mer counting

We use the *k*-mer counting tool Jellyfish (v2.2.4) [24] to generate the *k*-mers along with their counts from the input sequences for varying lengths (6-63). For each length, *k*-mers as keys and their counts as values have been stored as the outcome. Since the sequences of the species may come from either of the two strands of DNA, we aggregate the counts of the *k*-mers and their reverse complements. For instance, if the actual *k*-mer is TCGAC, then its count and the count of its reverse complement GTCGA are added and stored against the one that is lexicographically smaller.

### 2.3 Optimal *k*-mer length selection

In alignment-free approaches, a major issue is to determine optimal lengths of *k*-mer for phylogenomic analysis. It is a critical parameter for reaching the necessary resolution for genome distances in order to infer significant evolutionary relationships among species. Hence, a number of approaches to determine the optimal *k*-mer length from whole-genome sequences have been explored including cumulative relative entropy (CRE) [14], [25], [26], [27], [28]. Bai et al. [26] picked *k* based on the statistical power of identifying variations between two sequences. Recently a new tool, KITSUNE [29] also used a CRE based approach to select *k*-mer lengths for various data sets.

Here we use entropy for optimal *k*-mer length selection. Entropy is a measure of unpredictability or system disorder [30] which can determine disorder (e.g. variation between two DNA sequences). For small values of *k*, many *k*-mers will be present in a large number of sequences resulting in a low entropy whereas, for large values, each *k*-mer will be present in very few sequences which again will lead to a low entropy. For moderate values of *k*, the similarities of closely related species and dissimilarities of distantly related species will be captured and the entropy will be high. The higher the entropy, the more informative the *k*-mers. In this context the following equation has been used for entropy calculation:

$$H(X)=\sum_{i=1}^{m}\sum_{j=1}^{2}p(x_{ij})\log p(x_{ij})$$

Here, *m* is the number of *k*-mers and $p(x_{\text{i1}})$ and $p(x_{\text{i2}})$ denote the fraction of species where the *i*-th *k*-mer is present and absent respectively. For each *k*-mer length between 6 to 63, we calculate entropy using the above formula and then select the length for which the average entropy is the highest.

### 2.4 Distance calculation and phylogeny estimation

A number of approaches have been proposed previously to calculate distances from *k*-mer counts. The following three distance equations i.e. Euclidean squared distance [31], Mahalanobis distance [32] and fractional common *k*-mer count distance [33] have been used here for distance matrix formation:

**Squared Euclidean Distance:** $d^{E}(Q,S)=\sum_{i=1}^{4^{k}}{\left(q_{i}-s_{i}\right)}^{2}$

**Mahalanobis Distance:** $d^{M}(Q,S)=\sum_{i=1}^{4^{k}}{\left(q_{i}/\sigma_{i}-s_{i}/\sigma_{i}\right)}^{2}$

**Fractional Common *k*-mer Count Distance:**

$$d^{FC}(Q,S)=|\log(\epsilon +\sum_{i=1}^{4^{k}}(\min(q_{i},s_{i})/(\min(n,m)-k+1)))|$$

where, $q_{\text{i}}$ and $s_{\text{i}}$ are the frequency of the $i^{\text{th}}$ of $4^{\text{k}}$ possible substrings of length *k* in *Q* and *S* sequences respectively, $\sigma_{\text{i}}$ is the standard deviation, and *n* and *m* are the lengths of *Q* and *S* respectively.

And finally, phylogenetic trees are estimated from the constructed distance matrix using the widely used Neighbor Joining algorithm.

### 2.5 Missing region detection

Inference of phylogenetic trees from large-scale genomic data sets presents enormous computational and statistical challenges [34]. Furthermore, the presence of genomic rearrangements in the sequences makes sequence alignment difficult. While some of these challenges are circumvented by alignment-free approaches, missing regions in the input sequences of one or more species pose another challenge to alignment-free methods. We now turn to our method for detecting and filtering *k*-mers from missing regions without aligning the sequences.

**Figure 3:**
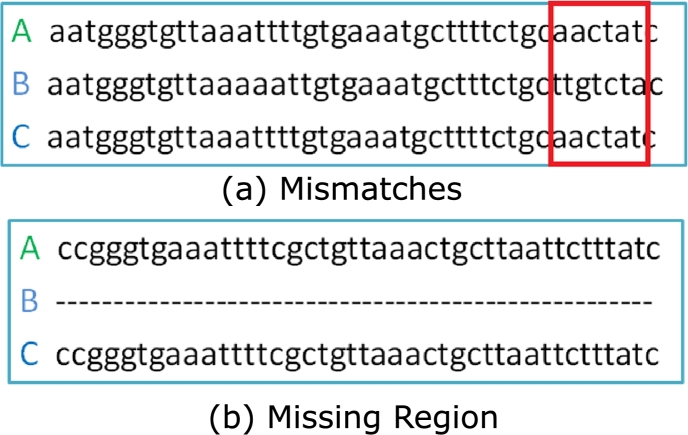
*k*-mers present in Sequence A but absent in Sequence B due to (a) mismatches (shown in red box) and (b) missing region.

Consider a *k*-mer which is present in one of the species but absent from some other species. This may be due to a portion missing from the sequence of the second species or it may be due to a substitution, insertion, or deletion of a small number of nucleotides. This is illustrated in Fig. 3. In both cases, Sequence A contains a *k*-mer which is not present in Sequence B. However, in Fig. 3(a), this is due to some mismatches (shown in the red box) whereas in Fig. 3(b), the entire region is missing from B.

We can distinguish between the two cases using the presence and absence of substrings of the *k*-mer of interest i.e. smaller *k*-mers in the sequence where the original *k*-mer is absent. If the *k*-mer is absent due to substitutions, small insertions or deletions, a large fraction of the small *k*-mers will be present in the sequence. However, if it is absent due to a missing region, many of the small *k*-mers will also be absent in that sequence. We can then use the following technique to detect *k*-mers corresponding to a missing region:

• The *k*-mers as well as their reverse complements that are not present in all of the species are marked and subdivided into small *k*-mers i.e. substrings of a smaller length.
• If the number of substrings of a *k*-mer that are absent in a species exceeds some threshold, the *k*-mer is deemed absent due to a missing region and is discarded.
• If the above count is below the threshold, the *k*-mer is assumed to be absent due to substitutions or indels and is retained for distance calculation.

The process is summarized in Algorithm 1. We use this algorithm to filter out *k*-mers likely to be due to missing regions. A distance matrix is then constructed with the new list of remaining *k*-mers and their frequencies.

**Algorithm 1:**
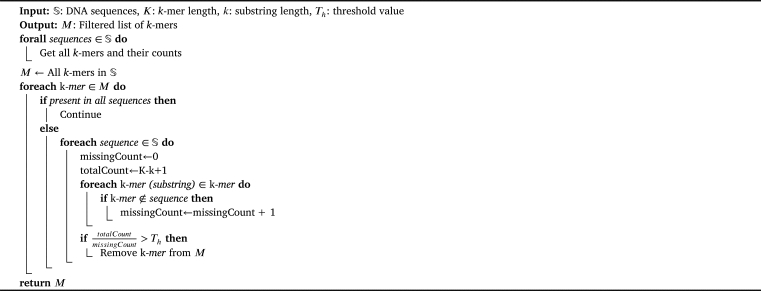
Filter *k*-mers.

## 3 Results

To assess the accuracy of our method, we construct distance matrices with and without filtering *k*-mers and construct the corresponding phylogenies for a number of real and simulated datasets, and compare them with reference trees using the Robinson–Foulds (RF) distance [35], which is based on the number of partitions of taxa implied by the first tree but not the second tree, and vice versa.

### 3.1 Datasets

We analyze our method on six real datasets. The first dataset is the 3 species dataset containing sequences downloaded from the NIH Intramural Sequencing Center (NISC) discussed previously. The second dataset, which was analyzed in [8], contains full mitochondrial genome sequences of 7 primates. The third dataset is a subset of the dataset examined in [36] containing 6 avian species. The other three data sets are part of the AFproject [7] which is an open platform for comparing different alignment-free methods. They include a dataset containing DNA sequences of 11 different species of mammals, assembled genomes of 6 *E.coli/Shigella* strains, and fully assembled genomes of 25 fish species [37] from the suborder Labroidei. For comparison with the reference tree using the RF distance, the reference tree for 6 avians is available at [36], the reference trees for 11 mammals, 6 E.coli/Shigellas and 25 fishes have been collected from the AFproject [7], [38], and the reference tree for seven primates has been obtained from [8].

### 3.2 Selection of the substring length and the threshold value

First, we experiment with the datasets to set the two parameters of the method - the length of the smaller *k*-mer (substring length *k*), and the threshold $T_{h}$ for filtering. We performed multiple sequence alignment of the 3 species and the 7 primates datasets using the tool *MAFFT* [39]. If the number of characters aligning to gaps is greater than one third of the *k*-mer length, then we label this portion as missing. The *k*-mers are thus labeled as from missing regions based on alignment, and used to calculate sensitivity and specificity.

**Figure 4:**
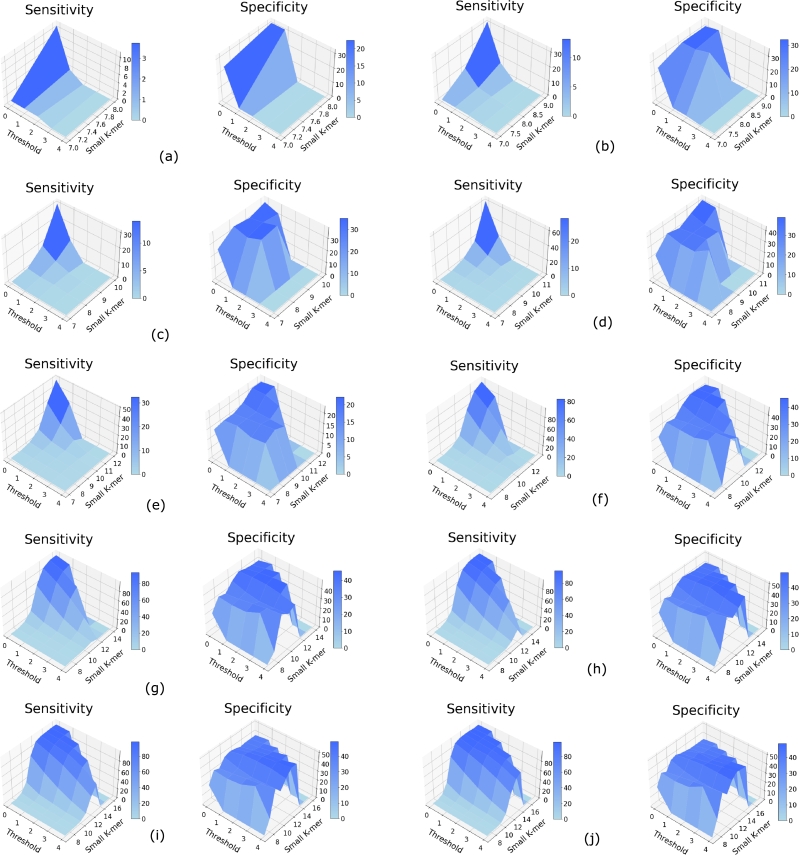
Sensitivity and specificity to detect *k*-mers corresponding to missing regions for the 3 Species dataset for varying small *k*-mer (substring) length and threshold values for *k*-mer lengths (a) 9, (b) 10, (c) 11, (d) 12, (e) 13, (f) 14, (g) 15, (h) 16, (i) 17, and (j) 18.

**Figure 5:**
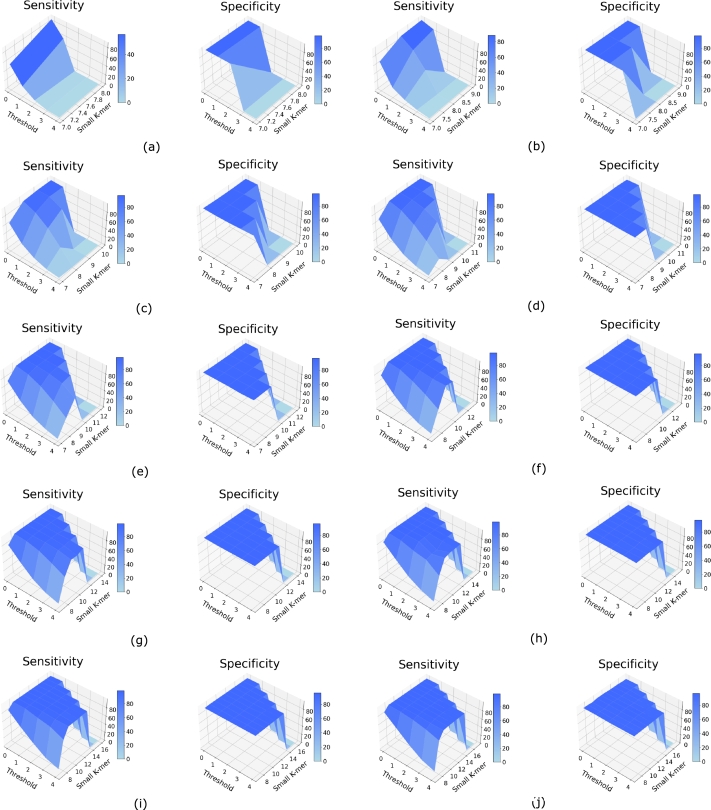
Sensitivity and specificity to detect *k*-mers corresponding to missing regions for the 7 Primates dataset for varying small *k*-mer (substring) length and threshold values for *k*-mer lengths (a) 9, (b) 10, (c) 11, (d) 12, (e) 13, (f) 14, (g) 15, (h) 16, (i) 17, and (j) 18.

Then for a range of large *k*-mer length *K* from 9 to 18, we varied *k* from 7 to the maximum substring length, and $T_{h}$ from 0 to 4 (defined below), and calculated the sensitivity and specificity of the method. They are shown in Figs. 4(a-j) and 5(a-j). We observe that as *k* increases, sensitivity generally increases. However, there is often a drop in specificity for high values of *k*. We also find that values of *k* less than 7 lead to inaccurate results (not shown), possibly due to the presence of small *k*-mers in other regions in such cases. Therefore, we use the following formula to set *k*:

$$k=\max(K-\left\lfloor \frac{K}{5}\right\rfloor ,7)$$

where *k* is the small *k*-mer and *K* is the selected length of *k*-mer for phylogeny estimation.

Figs. 4(a-j) and 5(a-j) also show how sensitivity and specificity changes for varying threshold $T_{h}$ values which is defined as:

$$T_{h}=\frac{\text{Total number of substrings of length}\;\text{k}}{\text{Number of missing substrings of length}\;\text{k}}$$

We find that both sensitivity and specificity are low for high values of the threshold and sensitivity is also low for very small values of $T_{h}$. Based on these observations we set $T_{h}=2$, i.e. if more than m/2 of the substrings are missing, we consider the large *k*-mer to be missing where *m* is the total number of substrings of length *k* given by

m=K−k+1.

### 3.3 *k*-mer length selection using entropy

**Figure 6:**
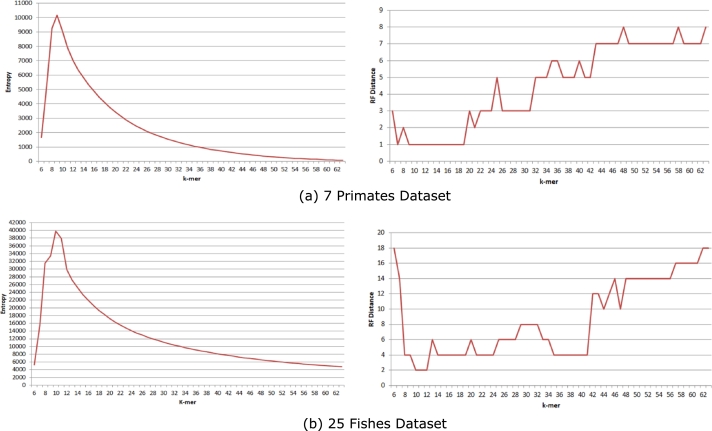
Selection of *k*-mer length from entropy values. Plots showing different entropy values and RF distances from varying values of *k* for (a) 7 Primates dataset and (b) 25 Fishes dataset.

As mentioned earlier, we use maximum entropy to select the length of the large *k*-mer used for phylogeny estimation. The entropy and the RF distances against *k*-mer lengths for the 7 Primates dataset and the 25 Fishes dataset have been shown in Fig. 6(a) and 6(b) respectively. We find that for the 7 Primates dataset, the highest entropy is at *k*-mer length of 9 which also gives the minimum RF distance of 1. Again, for the 25 Fishes dataset, both the highest entropy and the minimum RF distance are obtained for 10-mer. Similarly, for all the datasets we have analyzed, the minimum RF distance is achieved at the *k*-mer length with the highest entropy.

### 3.4 Assessment of distance metrics for phylogeny estimation

While estimating phylogenetic trees, fractional common *k*-mer count distance measure has performed better than the Euclidean squared and the Mahalanobis distances in the datasets we have analyzed. Among the six datasets mentioned earlier, though all of the distances show the same outcome for the 3 Species, 7 Primates and 6 *E.coli* datasets, fractional common *k*-mer count distance yields better results for the 6 avians, 25 Fishes and the 11 Mammals datasets (Table 1). Hence, we restrict our analysis to fractional common *k*-mer count distance measure in the remainder of the paper.

**Table 1:** Performance of distance metrics.

Datasets	RF Distance		
	Mahalanobis	Fractional Common *k*-mer Count	Euclidean
3 Species	2	2	2
7 Primates	1	1	1
25 Fishes	4	2	4
6 Avians	9	4	7
6 *E.coli*	5	5	5
11 Mammals	4	0	4

### 3.5 Results on real datasets

After setting the parameters of our method, we apply it to analyze the six real datasets described earlier. The results are summarized in Table 2. We observe that for the 3 Species dataset, the RF distance before filtering is 2 i.e. there is an error in the estimated tree if the tree is constructed using all *k*-mers. Our method is able to detect missing *k*-mers with 78% sensitivity and 49% specificity. In this case, when the *k*-mers are filtered using our method, the estimated tree matches the reference resulting in an RF distance of 0.

**Table 2:** The table shows the *k*-mer lengths, substring lengths, threshold values, sensitivity and specificity of the method to detect *k*-mers from missing regions, and the RF distances of the constructed phylogenies before and after filtering *k*-mers using the method for various datasets.

Datasets	*k*-mer length	Substring length	Threshold	Sensitivity	Specificity	RF Distance	
						Before Filtering	After Filtering
**3 Species**	16	13	2	78	49	2	0
**7 Primates**	9	8	2	60	98	1	1
**25 Fishes**	10	8	2	78	60	2	2
**6 Avians**	13	11	2	*	*	4	2
**6 *E.coli***	19	16	2	*	*	5	3
**11 Mammals**	15	12	2	*	*	0	0

* As the 6 Avians, 6 E.coli/Shigellas, and 11 Mammals datasets are too large to align, the sensitivity and specificity could not be calculated.

For the 7 Primates and the 25 Fishes datasets, the sensitivity and specificity values in Table 2 indicate that our method is again able to detect large fractions of the missing *k*-mers. For the avian and *E.coli* datasets, RF distances improve from 4 to 2, and from 5 to 3, respectively, after filtering. However, for the 7 primates and 25 fish datasets, the RF distances do not improve after filtering. This may be because our method was not able to filter all the missing *k*-mers. Another possible reason is that the error is due to other issues in addition to the missing regions. Finally, for the 11 Mammals dataset, the tree contains no errors before filtering which is also the case after filtering. For the avian, *E.coli* and mammals datasets, the sensitivity and the specificity could not be calculated as the sequences could not be aligned with MAFFT.

### 3.6 Simulation results

Finally, we perform a simulation study on the 7 Primates and the 25 Fishes datasets to thoroughly assess the effectiveness of our method in dealing with missing regions. From the sequences of each species in the two datasets, we remove regions of varying lengths from randomly chosen locations. First, we estimate phylogenies from the datasets containing missing regions without performing any filtering. Next, we filter out *k*-mers using our method and construct phylogenies with the remaining *k*-mers.

**Figure 7:**
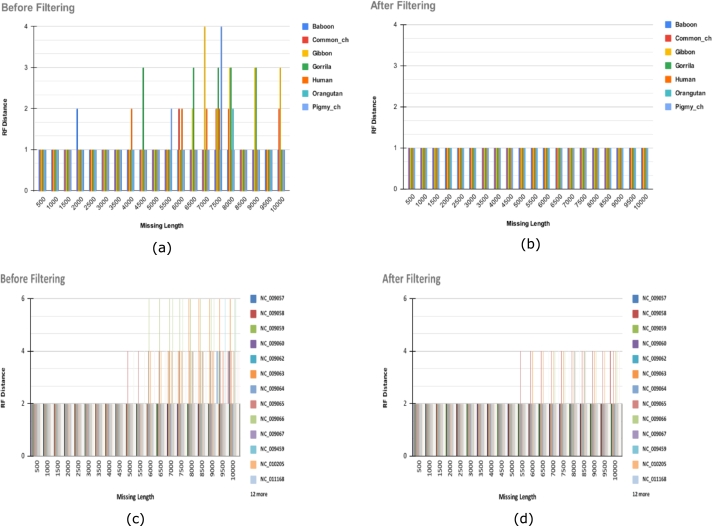
Comparison of RF distances before and after filtering (a) & (b) for 7 primates, and (c) & (d) for 25 fishes. Regions of varying lengths were removed from sequences of each species and RF distances of the estimated phylogenies were calculated.

Fig. 7 shows the RF distances against lengths of missing regions for the two datasets before and after filtering *k*-mers using our method. The phylogenies constructed from the 7 Primates and the 25 Fishes datasets had RF distances of 1 and 2, respectively, before introducing missing regions. We find that the introduction of missing regions leads to increases in RF distances in some cases. After filtering using our method, the RF distances generally decrease.

For the 7 Primates dataset, the RF distances increased to 2, 3, and 4 after the deletion of regions (Fig. 7(a). But after filtering, the RF distances decrease to 1 (Fig. 7(b) which is the value before the removal of regions. Similarly, for the 25 Fishes dataset, the RF distances show a general decreasing trend after filtering (Figs. 7(c) and 7(d)). However, in this case, the RF distances are sometimes not reduced to the levels before the introduction of missing regions in some instances.

## 4 Discussion

In this study, we introduced an alignment-free approach for detecting missing regions in *k*-mer count-based phylogeny estimation methods. Our method offers a promising solution to a key challenge in alignment-free phylogenetics, where missing data can significantly impact the accuracy of estimated phylogenies. Our results demonstrate the effectiveness of the proposed alignment-free approach in detecting missing regions. By leveraging counts of substrings of *k*-mers, our method successfully identifies large fractions of missing *k*-mers. This potentially improves the robustness and accuracy of alignment-free techniques in phylogeny estimation, which is particularly important in scenarios where alignment-based methods struggle to scale or accurately handle rearrangements.

While our method demonstrates promising results across a range of datasets, it has some limitations. In some instances, the method does not fully capture all missing regions, leading to residual errors in phylogenetic reconstructions. Furthermore, we observed that using substring length less than 7 sometimes leads to inaccurate results. This makes it difficult to apply our method to distantly related species. Future research should explore strategies to enhance the robustness of the method and address these limitations.

The findings of this study have significant implications for the field of phylogenetics. By providing a robust method for detecting missing regions in alignment-free phylogeny estimation, our approach contributes to more accurate reconstructions of evolutionary relationships. Looking forward, there is scope for further research to refine and extend our approach or to explore alternative strategies for detecting missing regions to overcome its limitations. Then eventually our method may be integrated into existing phylogeny estimation pipelines.

## 5 Conclusion

Alignment-free methods for phylogeny estimation are increasingly becoming popular as sequence alignment tools are difficult to scale to long sequences and especially in the presence of rearrangements. However, missing regions in the sequences, which are trivially detected during alignment, pose challenges in alignment-free phylogeny estimation. In this paper, we presented an alignment-free approach for missing region detection for *k*-mer count based alignment-free phylogeny estimation methods. Our method filters out *k*-mers that are likely to correspond to regions missing in one or more of the species using counts of substrings of the *k*-mers. We analyze real and simulated datasets and find that our method can detect and filter out large fractions of such *k*-mers, and generally lead to improvements in the estimated phylogenies. However, in some instances the estimated trees still contain errors. In the future, this may be investigated and the method can be modified accordingly. This approach may eventually be incorporated in pipelines for alignment-free phylogeny construction to improve robustness to missing regions.

## CRediT authorship contribution statement

**Rubyeat Islam:** Writing – review & editing, Writing – original draft, Visualization, Validation, Software, Resources, Methodology, Investigation, Formal analysis, Data curation, Conceptualization. **Atif Rahman:** Writing – review & editing, Writing – original draft, Validation, Supervision, Project administration, Methodology, Formal analysis, Data curation, Conceptualization.

## Declaration of Competing Interest

The authors declare that they have no known competing financial interests or personal relationships that could have appeared to influence the work reported in this paper.

## Data Availability

The datasets analyzed in this study are publicly available, and the sources have been referenced in the “Datasets” subsection under “Results”.
